# Overcoming function annotation errors in the Gram-positive pathogen *Streptococcus suis *by a proteomics-driven approach

**DOI:** 10.1186/1471-2164-9-588

**Published:** 2008-12-05

**Authors:** Manuel J Rodríguez-Ortega, Inmaculada Luque, Carmen Tarradas, José A Bárcena

**Affiliations:** 1 Departamento de Bioquímica y Biología Molecular, Universidad de Córdoba, 14071 Córdoba, Spain; 2 Departamento de Sanidad Animal, Universidad de Córdoba, Spain

## Abstract

**Background:**

Annotation of protein-coding genes is a key step in sequencing projects. Protein functions are mainly assigned on the basis of the amino acid sequence alone by searching of homologous proteins. However, fully automated annotation processes often lead to wrong prediction of protein functions, and therefore time-intensive manual curation is often essential. Here we describe a fast and reliable way to correct function annotation in sequencing projects, focusing on surface proteomes. We use a proteomics approach, previously proven to be very powerful for identifying new vaccine candidates against Gram-positive pathogens. It consists of shaving the surface of intact cells with two proteases, the specific cleavage-site trypsin and the unspecific proteinase K, followed by LC/MS/MS analysis of the resulting peptides. The identified proteins are contrasted by computational analysis and their sequences are inspected to correct possible errors in function prediction.

**Results:**

When applied to the zoonotic pathogen *Streptococcus suis*, of which two strains have been recently sequenced and annotated, we identified a set of surface proteins without cytoplasmic contamination: all the proteins identified had exporting or retention signals towards the outside and/or the cell surface, and viability of protease-treated cells was not affected. The combination of both experimental evidences and computational methods allowed us to determine that two of these proteins are putative extracellular new adhesins that had been previously attributed a wrong cytoplasmic function. One of them is a putative component of the pilus of this bacterium.

**Conclusion:**

We illustrate the complementary nature of laboratory-based and computational methods to examine in concert the localization of a set of proteins in the cell, and demonstrate the utility of this proteomics-based strategy to experimentally correct function annotation errors in sequencing projects. This approach also contributes to provide strong experimental evidences that can be used to annotate those proteins for which a Gene Ontology (GO) term has not been assigned so far. Function annotation correction would then improve the identification of surface-associated proteins in bacterial pathogens, thus accelerating the discovery of new vaccines in infectious disease research.

## Background

A crucial goal of whole-genome sequencing projects is the annotation of protein-coding genes [1]. Undoubtedly, genome sequencing projects are the major source of predicted proteins at the current time, and the function of gene products is generally assigned on the basis of the amino acid sequence alone by searching of homologous proteins in other organisms through similarity search engines such as BLAST [2,3]. Despite recent advances in computational ORFs prediction, a comprehensive annotation of protein-coding genes remains challenging, as fully automated annotation processes often lead to wrong prediction of protein functions [4], and therefore time-intensive manual curation is often essential. However, most of the millions of protein sequences currently being deposited to sequence databases will never be annotated manually [5].

A consequence of the overwhelming amount of sequence information is that only a small fraction of predicted proteins have their function experimentally validated, by means of actual cellular localization, activity, etc [6]. Even for the best studied organism, *Escherichia coli*, a large number of proteins have never been identified and characterised, and/or await unravelling of their biological role [7]. It is estimated that 40–50% of proteins from complete genomes remain "hypothetical", i.e., with unknown function [3]. Sequence similarity is an indicator of potential function, but it is not an absolute criterion for function assignment, so it must be combined with experimental evidences [8,9]. In addition, given that protein function is strongly dependent on subcellular localization (SCL), SCL prediction algorithms can also help by means of identifying sequence features such as signal peptides or transmembrane domains [10,11]. These aspects are particularly important when the aim is to select surface antigens for high-throughput vaccine development against pathogens [12]. Therefore, high-throughput experimental methods will become an important part of any genome annotation strategy, as a second phase after the necessary, but often insufficient, *in silico *automated prediction for elucidating protein function [13,14]. Mass spectrometry-based proteomics is a powerful approach for validating gene annotation and predicting protein function, as it analyses proteins directly, verifying putative gene products at the level of translation [15,16].

**Figure 1:**
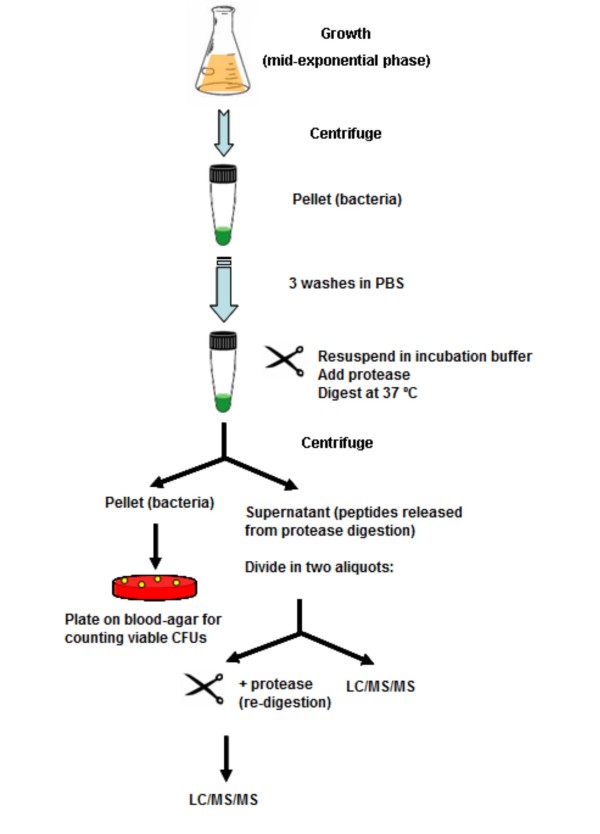
**Identification of surface proteins by shaving of the surface of live cells and LC/MS/MS analysis**. Bacteria were grown at mid-exponential phase and harvested by centrifugation. After washing in PBS, they were resuspended in incubation buffer and digested with a protease. Supernatants containing the released peptides were recovered, and an aliquot was re-digested with the same protease to make remaining large polypeptides more amenable to LC/MS/MS analysis. Pellets consisting of the shaved bacteria were plated onto blood-agar plates to test cell viability.

Here we present a new utility of a proteomics approach, which has proven to be very powerful for identifying new vaccine candidates against Gram-positive bacterial pathogens, focusing on surface proteomes [17], as a fast and reliable way to correct protein function annotation in complete sequencing projects. It consists of digesting the surface of live cultured cells with proteases in very mild conditions, to avoid cell lysis. The peptides released into the incubation buffer (the "surfome" or surface proteome) are analysed by LC/MS/MS (Figure 1), the identified proteins are validated by computational analysis and their sequences are inspected to correct possible errors in function prediction. As a model, the Gram-positive bacterium *Streptococcus suis *was used in this work, for which two completely annotated genomes (strains 05ZYH33 and 98HAH33) have recently been published [18]. This is an important pathogen associated with a wide range of diseases in pigs, including meningitis, septicaemia, pneumonia, endocarditis, and arthritis [19,20]. Human infection with *S. suis*, especially associated to serotype 2, has become a serious zoonosis and has been reported in many countries with intensive swine production [21,22]. More than 200 cases of infection have been described worldwide during the last decade, most of them from European and Asian countries. In July 2005, a large outbreak of human *S. suis *infection occurred in Sichuan province, China, and 53 people died due to toxic shock syndrome and meningitis [21,23]. The repeated intensive outbreaks of human *S. suis *infection have raised great public concern worldwide regarding this pathogen as an emerging zoonotic agent, as there is not an available vaccine against this microorganism. Therefore, any improvement in the information available on this organism at the functional genomics level would be highly valuable for researchers in the fight against this pathogen.

## Results and discussion

### Validation of subcellular location by combining proteomics with computational analysis

Treating the cells with two different proteases (trypsin, which cleaves specifically after lysine or arginine residues; and proteinase K, a protease that cleaves peptide bonds unspecifically, in conditions mild enough to avoid a complete degradation of peptides into single amino acids), we identified 28 proteins (Table 1 and Additional File 1), all of them corresponding to the four categories of surface proteins of Gram-positive bacteria [24]: i) LPXTG-cell wall proteins, containing a peptidoglycan-anchoring motif in the *C*-terminus of the protein, the LPXTG motif; ii) lipoproteins, linked to the underlying plasma membrane through a lipid covalently bound at their *N*-terminus; iii) secreted proteins, which can bind to the surface by charge/hydrophobic interactions; and iv) membrane proteins, embedded in the plasma membrane underlying the wall through at least one transmembrane helix (TMH). The treatments did not affect cell survival (Table 2) and the absence of peptides from cytoplasmic proteins was total, as an indication that the integrity of the wall had not been affected.

**Table 1:** Proteins identified by LC/MS/MS

***Gene locus*, Annotated protein function**	**Predicted subcellular localization**	**Prediction algorithm**
*ssu05_0753*, MRP	Cell wall	PSORTb v 2.0
*ssu05_1982*, Subtilisin-like serine protease	Cell wall	PSORTb v 2.0
*ssu05_1968*, DNA nuclease	Cell wall	PSORTb v 2.0
*ssu05_0196*, hypothetical protein SSU05_0196	Cell wall	PSORTb v 2.0
*ssu05_1311*, hypothetical protein SSU05_1311	Cell wall	PSORTb v 2.0
*ssu05_0965*, agglutinin receptor	Cell wall	PSORTb v 2.0
*ssu05_2064*, Type II secretory pathway, pullulanase PulA and related glycosidases	Cell wall	PSORTb v 2.0
*ssu05_1371*, Ribonucleases G and E	Cell wall	PSORTb v 2.0
*ssu05_1295*, hypothetical protein SSU05_1295	Cell wall	PSORTb v 2.0
*ssu05_0214*, ABC-type xylose transport system, periplasmic component	Cell wall	PSORTb v 2.0
*ssu05_1663*, Methyl-accepting chemotaxis protein	Cell wall	PSORTb v 2.0
*ssu05_0473*, Ribonucleases G and E	Cell wall	PSORTb v 2.0
*ssu05_0700*, ATPase (PilT family)	Lipoprotein	LipoP
*ssu05_1083*, Uncharacterized ABC-type transport system, periplasmic component/surface lipoprotein	Lipoprotein	LipoP
*ssu05_2133*, ABC transporter substrate-binding protein – maltose/maltodextrin	Lipoprotein	LipoP
*ssu05_0513*, Membrane-fusion protein	Membrane	TMHMM
*ssu05_1022*, hypothetical protein SSU05_1022	Membrane	TMHMM
*ssu05_1635*, Predicted xylanase/chitin deacetylase	Membrane	TMHMM
*ssu05_1354*, Cell division protein FtsI/penicillin-binding protein 2	Membrane	TMHMM
*ssu05_1509*, Negative regulator of septation ring formation	Membrane	TMHMM
*ssu05_2173*, LysM repeat protein	Membrane	TMHMM
*ssu05_1579*, Ammonia permease	Membrane	PSORTb v 2.0, TMHMM
*ssu05_1292*, Phosphoglycerol transferase and related proteins, alkaline phosphatase superfamily	Membrane	PSORTb v 2.0, TMHMM
*ssu05_1380*, ABC-type antimicrobial peptide transport system, permease component	Membrane	TMHMM
*ssu05_1282*, Predicted membrane protein	Membrane	PSORTb v 2.0, TMHMM
*ssu05_0332*, hypothetical protein SSU05_0332	Secreted	SignalP
*ssu05_0811*, Subtilisin-like serine protease	Secreted	SignalP
*ssu05_1682*, extracellular serine protease	Secreted	SignalP

Surface proteins identified after protease treatment of cultured live cells of *Streptococcus suis *serotype 2, strain 235/02. The table reports: 1) the accession number of the genes encoding the identified proteins, 2) the predicted cellular localization of the proteins, 3) the algorithms used for such predictions.

**Table 2:** Survival of bacterial cells after protease treatment

**Treatment**	**CFUs (× 10^8 ^cells/ml)**	**Statistical significance ^a^**
Control	2.96 ± 0.55	-
Trypsin	2.66 ± 0.21	NS
Proteinase K	2.86 ± 0.31	NS

Counting of CFUs (colony-forming units), after plating on THY plates supplemented with 5% sheep blood, *Streptococcus suis *serotype 2, strain 235/02 treated with trypsin or proteinase K for digestion of surface proteins of live cells. No protease was used in the controls.

^a ^Statistical significance was calculated by applying the Student's *t*-test (*P *< 0.05) when comparing treatments to the control: NS, not significant.

The identified proteins were checked by computational analysis. As a first approach, we used PSORTb v 2.0, which has been described to be the best subcellular-location prediction algorithm for bacteria because its high precision and recall [25]. However, when this algorithm was unable to return a confident prediction, feature-based methods were employed for searching exporting or retention motifs towards the outside and/or the surface of the cell. In addition, the primary sequences of the PSORTb-predicted cell wall proteins were manually inspected in search of the cell-wall sorting signal that characterises those covalently bound to the peptidoglycan (the LPXTG motif followed by a hydrophobic sequence that constitutes a transmembrane region, plus a short positively charged coil, at the *C*-terminus): when it was not present, the previously mentioned feature-based algorithms were applied.

**Figure 2:**
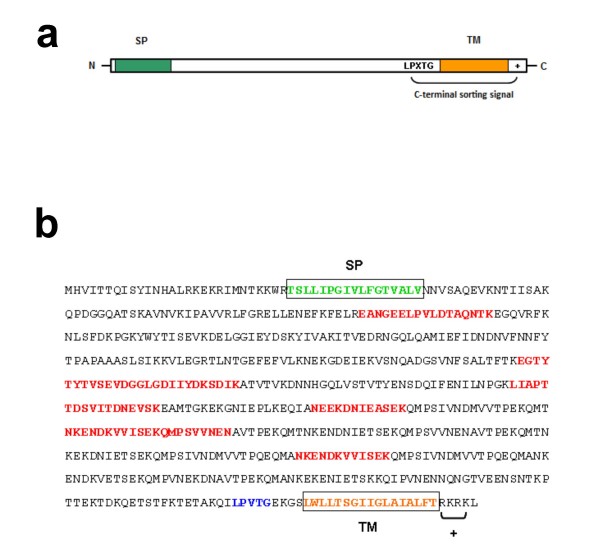
**Sequence pattern of LPXTG-anchoring cell wall proteins and identification of Ssu05_1371**. a) Structure of the primary sequence of cell-wall anchored proteins. They have the following elements: a signal peptide (SP) at the *N*-terminus and, at the *C*-terminus, the consensus sequence LPXTG for its recognition by a sortase, an enzyme that cleaves between T and G residues and binds the mature protein to the peptidoglycan layers of the cell wall. Following the LPXTG sequence, there is a hydrophobic region for transmembrane spanning (TM) of the immature form, and after, a short positively charged tail (+). b) Protein Ssu05_1371 shows the typical structure of a cell-wall protein. In red bold, sequence coverage by identified peptides by proteomics is shown (see Additional File 1).

Of the identified proteins, 12 were classed into the cell wall category (out of 19 predicted in the genome, 63%), all of them being predicted by PSORTb as belonging to this group and having the cell-wall sorting signal (Figure 2a). This is expected since the most abundant and exposed surface proteins in Gram-positive bacteria belong to this category [24].

Three proteins (out of 36 predicted in the genome, 8.3%) were classed as lipoproteins. For two of them (those coded by the loci *ssu05_1083 *and *ssu05_2133*), PSORTb did not produce a prediction (this algorithm does not predict type-II signal peptides [25,26]). For the other one (that encoded in the *ssu05_0700 *locus), PSORTb returned the result "extracellular". However, LipoP revealed the presence of a SPaseII cleavage site between positions 22 and 23, so this protein was classified in the lipoprotein group.

Ten proteins were classified as membrane proteins (out of 506 predicted in the genome, 2%), but for only 3 of them, PSORTb returned this prediction (those coded by the genes *ssu05_1579*, *ssu05_1282 *and *ssu05_1292*). For the sequence encoded in the locus *ssu05_1022*, PSORTb predicted a cell-wall protein, despite the absence of the cell-wall sorting signal. Moreover, TMHMM predicted 3 TMHs for this protein, all of them located near the *N*-terminus of the sequence. The same occurred for protein Ssu05_2173, which has, according to TMHMM, one TMH at the *N*-terminus of its sequence. It must be also highlighted that, for protein Ssu05_1509, PSORTb predicted a cytoplasmic location, but TMHMM revealed a TMH (neither SignalP nor LipoP returned a prediction for this protein). In summary, the possession of TMHs in these 10 proteins was confirmed by TMHMM. As described by Rey et al [10], correct identification of membrane proteins by PSORTb is not very confident when these have one or two helices. However, PSORTb reveals itself as a very powerful tool for detecting both LPXTG-cell wall proteins and membrane proteins with three or more TMHs [25]. Finally, 3 proteins were classed as extracellular/secreted proteins (out of 25 predicted in the genome, 12%), possessing a type-I signal peptide (substrate for SPaseI) according to SignalP. For two of these proteins (Ssu05_0332 and Ssu05_1682), PSORTb did not return any prediction. For the remaining protein (Ssu05_0811), the PSORTb prediction was "cell wall", but this sequence lacked the cell-wall sorting signal. However, the PSORTb prediction in this case may not necessarily be incorrect. In Gram-positive organisms, the main cell-wall proteins are those covalently linked to the peptidoglycan, containing the consensus sequence LPXTG (or some variation of this motif, especially in pilin proteins [27]) followed by the cell-wall sorting signal (Figure 2a). But many proteins secreted through the type I secretion system are bound to the cell surface via non-covalent interactions, including choline-binding domains, LysM domains, GW-modules, and others [26]. Then, the same could be considered for protein Ssu05_2173, which has been annotated as a LysM protein: PSORTb predicts it to be a cell-wall protein, whereas feature-based methods did not agree in their predictions: SignalP predicted a signal peptide, with a cleavage site in 38–39, and TMHMM predicted a TMH in residues 7–26. It is well established that it is not always easy to distinguish between both type-I signal peptides and TMHs [25,26,28]. At least, what is clear for protein Ssu05_2173 is the fact that it has some exporting or retention signal towards the exterior or the cell surface. Nevertheless, we cannot rule out that some of the identified proteins may be localized to various compartments: e.g., some secreted proteins, in addition to be soluble outside the cell, are partially bound to the surface by non-covalent interactions. Recently, a new multi-component subcellular-location predictor for bacterial proteins has been described: LocateP [26]. It can distinguish 7 SCLs in Gram-positive bacteria. When applied to our dataset, it returned the same predictions as showed in Table 1, except for proteins Ssu05_0811 and Ssu05_1682, for which LocateP returned a " [membrane] *N*-terminally anchored" prediction (PSORTb returned unknown predictions for both of them). For protein Ssu05_1509, the prediction was also " [membrane] *N*-terminally anchored", in agreement with TMHMM. As explained above, this discrepancy could be due to the fact that these proteins may be localized at more than one compartment; and also that type-I signal peptides and TMHs are sometimes difficult to distinguish.

The bacterial surface is a fundamental site of interaction between cell and its environment [24]. Surface proteins constitute a diverse group of molecules involved in adhesion to and invasion of host cells, signalling, defence, toxicity, etc. Hence, they are potential targets for drugs aimed at preventing bacterial infections and diseases [29]. Moreover, because surface proteins are likely to interact with the host immune system, they may become components of effective vaccines [30]. Here, we aimed to identify surface-attached proteins in the Gram-positive pathogen *Streptococcus suis *by a proteomics approach consisting of shaving the bacterial surface with proteases [17]. In principle, this approach could be used and optimised for a wide range of biological systems.

Computational analyses confirm this strategy in terms of the quality of identifications, i.e., exclusively proteins with exporting or retention signals towards the outside and/or the surface of the cell [28]. Reciprocally, this proteomic approach validates the prediction algorithms, as it allows to identifying surface proteins as potential vaccine candidates, some of them being hypothetical proteins not found before experimentally. Moreover, proteins for which predictions by computational analyses disagree (e.g. Ssu05_1509) are confirmed to be surface-located by this proteomic approach, thus showing that the experimental validation of prediction program results can be useful for improving prediction algorithms. Therefore, these results illustrate the complementary nature of laboratory-based and computational methods to examine in concert the localization of a set of proteins in the cell, thus helping to focus research projects on the effective discovery of vaccine candidates [10,11].

### Functional annotation of identified proteins

For two identified proteins, coded by the loci *ssu05_1371 *and *ssu05_0473*, and both annotated as "ribonucleases G and E", the assigned functions were not in agreement with their primary sequences. When examining these sequences at the amino acid level, they showed the canonical architecture of the cell-wall attached proteins, as shown in Figure 2a. Figure 2b shows the sequence of Ssu05_1371 with the elements that define a typical LPXTG-anchoring cell wall protein, and its coverage by peptides identified after protease treatment of the surface of live cells.

Ribonucleases G and E are a family of endonucleases involved in RNA processing: their cellular localization is, therefore, cytoplasmic. They are mainly present in Gram-negative bacteria, and rarely detected in Gram-positive organisms [31] (Additional File 2). In fact, Ssu05_1371 is not similar to any of the proteins annotated as ribonucleases G and E in the databases. However, similarity (83% identity) was found to protein Sao from *Streptococcus suis *(GenBank accession number AY864331, and named "surface protein SP1" in the not yet fully annotated strain 89/1591), which has been shown to be surface-located and also to protect immunised animals against infection [32,33]. Moreover, Ssu05_1371 was highly similar to other streptococcal proteins that have characteristic functions attributed to surface proteins, as binding to the extracellular matrix (ECM) of the host cells (Additional File 3). These proteins are known generally as adhesins or, more specifically, MSCRAMMs (Microbial Surface Components Recognising Adhesive Matrix Molecules) when they bind ECM proteins such as fibronectin or collagen [34,35]. Adhesins mediate adherence to host cells or tissues during the first steps of infection [36].

**Figure 3:**
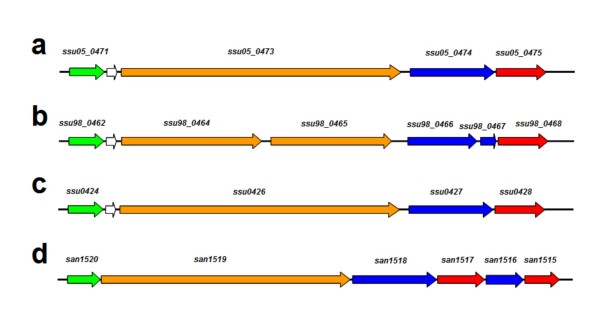
**Pilus islands in *Streptococcus suis***. Genomic organization of the pilus islands in strains 05ZYH33, 98HAH33 and P1/7 (a, b and c, respectively) and comparison to pilus island 2b (PI-2b) of *Streptococcus agalactiae *COH1 (d). These are composed of a gene coding for a signal peptidase I (green arrows), a major pilin protein containing the LPXTG-anchoring motif (orange arrows) that would constitute the pilus backbone, one or two ancillary proteins also containing the LPXTG motif (blue arrows) and one or two class C sortases (red arrows). Other genes within the clusters are shown by white arrows.

The locus *ssu05_0473 *codes for a large protein of 1603 amino acids, showing the structure of a cell-wall protein with the LPXTG motif (Figure 2a). This locus is in a region that is analogous to the pilus island 2b (PI-2b) from *Streptococcus agalactiae *COH1 [27] (Figure 3), which has recently been found in the draft genome of *S. suis *P1/7 [37]. Pili are filamentous structures that, in Gram-positive organisms, serve to adhere and invade host cells [38]. In Gram-positive bacteria, the genes for pili occur in clusters, which may constitute mobile genetic elements [27]. Protein Ssu05_0473 was not identified by trypsin digestion and this is reminiscent of the *S. pyogenes *pilin proteins which were previously named "T antigens" (for "trypsin resistant") [17,27,38]. The identification of protein Ssu05_0473 was achieved by two peptides after proteinase K treatment (Additional File 1). Ssu05_0473 would constitute the pilus backbone of *S. suis*, as the protein SAN_1519 for the type-2b pilus in *Streptococcus agalactiae *does (Figure 3). This is in agreement with the finding that the pilin proteins of *S. pyogenes *were identified only after proteinase K digestion [17], thus indicating that these proteins are more recalcitrant, maybe because of their particular folding and/or assembly when taking part in the pilus [39].

**Figure 4:**
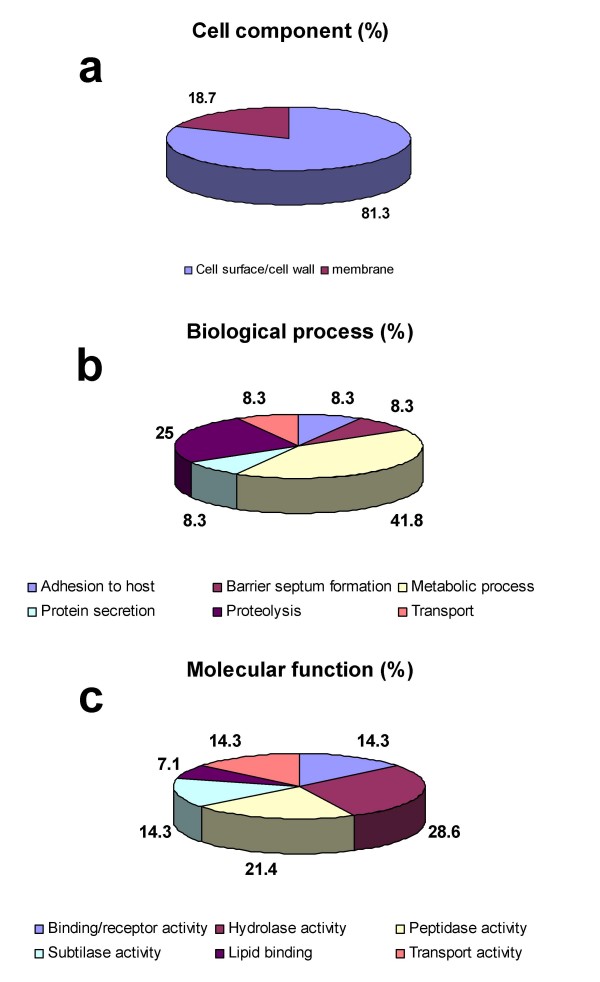
**Gene Ontology (GO) annotation of identifiedproteins**. The graphs show the percentages of corresponding GO terms on the total number of annotated proteins. 16 out of 28 proteins (57%) were annotated for "cell component" (a), 12 (43%) for "biological process" (b) and 14 (50%) for "molecular function" (c).

To functionally annotate the identified proteins, we mapped them to GO at the three levels: cell component, biological process and molecular function (Figure 4). For cell component, 12 proteins out of 28 (43%) had not a GO annotation; 16 out of 28 (57%) were not annotated for biological process, and 14 (50%) lacked a GO annotation at molecular function level (Additional File 4). However, the cell component GO annotation revealed that all the GO annotated proteins were located at the surface or at the membrane; for terms referring biological process, six different processes were GO annotated. Finally, the GO annotation for molecular function showed that hydrolase activity was predominant among those reported (64.3%), as it implies not only the term "hydrolase activity" (GO:0016787), but also its child term "peptidase activity" (GO:0008233) and its grandchild "subtilase activity" (GO:0004289). Many surface and secreted proteins are known virulence factors with hydrolase activity: hyaluronidases, neuraminidases, cysteine proteases, peptidases, hydrolytic enzymes degrading polysaccharides of extracellular matrices, etc [24,40-43].

Automatic annotation of protein functions often results in different types of errors, some of which can be overcome by combining manual annotation and experimental evidence [16]. Mass spectrometry-based methods can validate and correct assignments from automated function annotations [6]. We show a new utility of the proteomics approach here applied, which is especially suitable for a fast and reliable selection of surface proteins as vaccine candidates. Such a new utility provides an experimental support for the rapid correction of possible annotation errors in sequencing projects. In fact, two proteins with a wrongly attributed cytoplasmic function were identified in the set of "surfome" peptides, and both had the typical LPXTG-cell wall anchoring protein structure, thus indicating that their function predictions had been uncorrectly assigned. One of them (Ssu05_1371), previously reported as Sao protein, has been demonstrated to be surface-located by immunomicroscopy and also to protect mice against infection. Moreover, in our analyses, this protein was also found in all 5 strains analysed so far (results not shown), thus indicating that it may be a good candidate for vaccine development. The other one (Ssu05_0473), a putative pilin protein, is also an adhesin: pili are major structures participating in the adherence to and invasion of host cells [39,44,45]. In addition, the pilus proteins also have a strong protective capacity in animal models [46]. This experimental approach is also validated by the GO annotations: in our case, all the cellular component GO annotations confirmed what was intended to obtain, i.e. surface-associated proteins. Moreover, this approach provides direct experimental evidence for annotation to a GO cellular component term(s), which is an improvement in the GO annotations for these proteins, whose primary inferred function by electronic annotation is based on InterPro motif searching (Additional File 4). Such an improvement of experimental GO annotation would facilitate a higher accuracy of prediction programs. This problem has been also addressed by the DDF-MudPIT strategy [47], but this method has not been tested in prokaryotes.

### Overcoming false positives from cytoplasmic contamination

One of the most controversial aspects of experimental approaches to identify surface proteins is that, very often, cross-contamination by cytoplasmic proteins is found (sometimes in large amounts) when subcellular fractionation by classical biochemical methods are used. Highly abundant cytoplasmic proteins, like enolase, elongation factors, GroEL/ES chaperonins or ribosomal proteins are frequently observed contaminants in membrane/surface protein/secretome fractions; however, many studies do not address this fact. This leads to false positives in the resulting datasets [10]. The most plausible hypothesis to explain this is the occurrence of lysis in the culture, *prior *to obtaining the protein/peptide fraction.

In the present study, all the identified proteins had exporting or retention signals towards the outside and/or the surface of the cell [28], thus indicating the absence of contamination by cytoplasmic proteins. Protease treatment did not impair cell integrity, in terms of viable cells (Table 2). We can then infer that we have captured the population of peptides belonging to protein outer domains long enough to be actually exposed at the cell surface without causing cell lysis.

The approach here used has been demonstrated to work well with Gram-positive organisms, as they have a rigid cell wall that could make them more resistant to lysis than other types of cells. In the analyses here presented for the studied strain 235/02, no lysis took place, that is: neither cytoplasmic proteins were identified, nor lower viable counts for protease-treated cells were found. In the genus *Streptococcus*, the ease to lyse can vary among species, and factors causing this phenomenon are not yet completely understood. An explanation is the production of peptidoglycan (murein) hydrolases, which are enzymes degrading the cell wall, especially important in the pneumococcus, which produces several of these proteins, called autolysins [48]. Autolysis in *S. pneumoniae *seems to be a phenomenon by which a subset of the bacterial population die during the competence status, which is advantageous for surviving cells, as many virulence factors that help invasion are released [48,49]. Using strains with mutations in genes coding for these autolysins could help to solve the lysis problem. However, it cannot be ruled out that anchorless surface proteins reach and attach the microbial surface by yet unknown mechanisms [50]. Further research is needed to throw light upon some dark zones of this important issue, that is, the existence of moonlighting proteins [51].

The proteomic approach here applied, combined with computational analyses, is an optimal way to address these problems.

## Conclusion

We report a high-throughput proteomics strategy to experimentally validate and correct function annotation errors from predictions made by computational analysis. Proteins with wrongly predicted functions present in the experimentally determined surface proteome are revisited and their sequences manually inspected. Function annotation correction would then lead to new discoveries, thus accelerating the discovery of new vaccines in infectious disease research, to improving the identification of surface-associated proteins in bacterial pathogens. In this work, we have shown that two putative new adhesins of *Streptococcus suis *have been unmasked; among them, an unnoticed putative component of the pilus. This strategy would also help to identify and characterise, when the occurrence of lysis is controlled, the moonlighting proteins, and would them differentiate from actual cytoplasmic contamination.

## Methods

### Bacterial strains and growth

*Streptococcus suis *serotype 2, strain 235/02, isolated from an infected pig in Córdoba, Spain in 2002, was grown in Todd-Hewitt broth supplemented with 0.5% yeast extract (THY) at 37°C and 5% CO_2_, until an OD_600 _of 0.25 (mid-exponential phase) was reached.

### Surface digestion of live cells and viability assays

One hundred ml of bacteria from mid-exponential growth phase (corresponding to approximately 10^10 ^cells at OD_600 _= 0.25) were harvested by centrifugation at 3,500 × *g *for 10 min at 4°C, and washed three times with PBS. Cells were resuspended in 0.8 ml of incubation buffer consisting of PBS/30% sucrose (pH 7.4 for trypsin digestion and pH 6.0 for proteinase K digestion). Proteolytic reactions were carried out with trypsin (Promega) at 10 μg/ml or proteinase K (Sigma) at 5 μg/ml, for 20 min at 37°C. Controls were carried out without adding any enzyme. The digestion mixtures were centrifuged at 3,500 × *g *for 10 min at 4°C, and the supernatants (containing the peptides and large poplypeptides not fully digested) were filtered using 0.22-μm pore-size filters (Millipore). An aliquot of each digestion reaction was re-digested each one with the same enzyme and concentration, trypsin digestion for 2 h and proteinase-K digestion for 20 min. Protease reactions were stopped with formic acid at 0.1% final concentration. Before analysis, salts were removed by using commercial mini-cartridges HLB-Oasis (Waters) and then eluting the peptides with increasing concentrations of acetonitrile, according to manufacturer's instructions. Peptide fractions were concentrated with a vacuum concentrator (Eppendorf), and kept in low-binding tubes at -20°C until further analysis. Viability of treated and non-treated bacteria with proteases was assayed by counting CFUs (colony-forming units) in THY plates containing 5% defibrinated sheep blood.

### LC/MS/MS analysis

All analyses were performed with a Surveyor HPLC System in tandem with a Finnigan LTQ mass spectrometer (Thermo Fisher Scientific, San Jose, USA) equipped with nanoelectrospray ionization interface (nESI). The separation column was 150 mm × 0.150 mm ProteoPep2 C18 (New Objective, USA) at a postsplit flow rate of 1 μl/min. For trapping of the digest a 5 mm × 0.3 mm precolumn Zorbax 300 SB-C18 (Agilent Technologies, Germany) was used. One fourth of the total sample volume, corresponding to 5 μl, was trapped at a flow rate of 10 μl/min for 10 minutes and 5% acetonitrile/0.1% formic acid. After that, the trapping column was switched on-line with the separation column and the gradient was started. Peptides were eluted with a 60-min gradient of 5–40% of acetonitrile/0.1% formic acid solution at a 250 nl/min flow rate. All separations were performed using a gradient of 5–40% solvent B for 60 minutes. MS data (Full Scan) were acquired in the positive ion mode over the 400–1500 m/z range. MS/MS data were acquired in dependent scan mode, selecting automatically the five most intense ions for fragmentation, with dynamic exclusion set to on. In all cases, a nESI spray voltage of 1.9 kV was used.

### Database searching and protein identification

Search and identification of peptides were performed using in batch mode the raw MS/MS data with a licensed version of MASCOT, in a non-redundant local database containing the 2,185 proteins derived from the complete genome sequence of *Streptococcus suis *strain 05ZYH33 (RefSeq NC_009442 downloaded from , NCBInr version 20071013). The MASCOT search parameters were: (i) species, *Streptococcus suis *strain 05ZYH33; (ii) allowed number of missed cleavages (only for trypsin digestion), 4; (iii) variable post-translational modifications, methionine oxidation, and deamidation of asparagine and glutamine residues; (iv) peptide mass tolerance, ± 500 p.p.m.; (v) fragment mass tolerance, ± 0.6 Da and (vi) peptide charge, from +1 to +4. The score thresholds for acceptance of protein identifications from at least one peptide were set by MASCOT as 19 for trypsin cleavage and 34 for proteinase K digestion. All spectra corresponding to positive identifications or near the thresholds were manually inspected.

### Bioinformatic analysis of protein sequences

Computational predictions of subcellular localization were carried out using the web-based algorithm PSORTb v 2.0 [52]. Feature-based algorithms were also used to contrast PSORTb predictions, especially when it returned an "unknown" output: TMHMM 2.0 [53] for searching transmembrane helices; SignalP 3.0 [54] for type-I signal peptides: those proteins containing only a cleavable type-I signal peptide as featured sequence were classed as secreted; LipoP [55] for identifying type-II signal peptides, which are characteristic of lipoproteins. Similarity searches were carried out using the BLAST suite [56]. Gene Ontology (GO) annotations were retrieved using the AmiGO browser . Additional information on protein families, motifs, predictions of subcellular localization and status of the protein was retrieved from UniProt Knowledgebase .

## Authors' contributions

MJRO. performed the experiments, analysed the data and wrote the manuscript. JAB provided support to the project. IL and CT provided the *Streptococcus suis *strains. All the authors contributed to research and experimental design and discussed the results and the manuscript.

## Supplementary Material

Additional file 1 **Peptides identified by LC/MS/MS**. The Excel document contains the peptides identified for all the proteins reported in this study after trypsin and proteinase K treatment of *Streptococcus suis *strain 235/02. Click here for file

Additional file 2 **Proteins annotated as ribonucleases G and E in bacteria**. The Word file contains a list of all the proteins predicted as ribonucleases G and E in bacteria, according to the non-redundant UniProt Knowledgebase. Entries are sorted by alphabetical order of UniProt codes. Click here for file

Additional file 3 **Proteins with similarity to Ssu05_1371**. The Word file contains a list of proteins from Gram-positive organisms showing significant similarity to Ssu05_1371 through BLAST search. All the proteins found have the cell wall-anchoring LPXTG motif. Click here for file

Additional file 4 **GO annotations of identified proteins**. The Excel file contains the gi identifiers, Uniprot codes, GO annotations and GO evidence codes for the proteins coded in the reported loci and identified in this study. Click here for file
