# Supplementary material for: Overcoming function annotation errors in the Gram-positive pathogen Streptococcus suis by a proteomics-driven approach
Source: BMC Genomics. 2008 Dec 5;9:588. doi: 10.1186/1471-2164-9-588 (PMC2613929; doi:10.1186/1471-2164-9-588)
Supplement: Additional file 2 — Proteins annotated as ribonucleases G and E in bacteria. The Word file contains a list of all the proteins predicted as ribonucleases G and E in bacteria, according to the non-redundant UniProt Knowledgebase. Entries are sorted by alphabetical order of UniProt codes. [file 1471-2164-9-588-S2.doc]

List of all the proteins predicted as ribonucleases G and E in bacteria, according to the non-redundant UniProt Knowledgebase (<http://www.uniprot.org/>). Entries are sorted by alphabetical order of UniProt codes. Gene Ontology annotations of functions for this class of proteins are as follows: GO:0005737; Cellular component: cytoplasm (inferred from electronic annotation from InterPro). GO:0004540; Molecular function: ribonuclease activity (inferred from electronic annotation from InterPro). GO:0003723; Molecular function: RNA binding (inferred from electronic annotation from InterPro). GO:0006396; Biological process: RNA processing (inferred from electronic annotation from InterPro).

| UniProt code | Name | Organism | Subcellular localization |
| --- | --- | --- | --- |
| A0YEU6_9GAMM | Ribonucleases G and E {GENE:ORFNames=GP2143_00342} | marine gamma proteobacterium HTCC2143 | Cytoplasm |
| A2VQQ1_9BURK | Ribonucleases G and E {GENE:ORFNames=BCPG_00263} | Burkholderia cenocepacia PC184 | Cytoplasm |
| A2VWK8_9BURK | Ribonucleases G and E {GENE:ORFNames=BCPG_02416} | Burkholderia cenocepacia PC184 | Cytoplasm |
| A2W8K6_9BURK | Ribonucleases G and E {GENE:ORFNames=BDAG_01013} | Burkholderia dolosa AUO158 | Cytoplasm |
| A2WAK3_9BURK | Ribonucleases G and E {GENE:ORFNames=BDAG_01742} | Burkholderia dolosa AUO158 | Cytoplasm |
| A2WBK9_9BURK | Ribonucleases G and E {GENE:ORFNames=BDAG_02112} | Burkholderia dolosa AUO158 | Cytoplasm |
| A3JIF1_9ALTE | Ribonucleases G and E {GENE:ORFNames=MELB17_08671} | Marinobacter sp. ELB17 | Cytoplasm |
| A3JK77_9ALTE | Ribonucleases G and E {GENE:ORFNames=MELB17_07174} | Marinobacter sp. ELB17 | Cytoplasm |
| A4TUF8_9PROT | Ribonucleases G and E {GENE:ORFNames=MGR_0225} | Magnetospirillum gryphiswaldense | Cytoplasm |
| A5D406_PELTS | Ribonucleases G and E {GENE:Name=CafA; OrderedLocusNames=PTH_0829} | Pelotomaculum thermopropionicum | Cytoplasm |
| A5GIN3_SYNPW | Ribonucleases G and E {GENE:Name=cafA; OrderedLocusNames=SynWH7803_0372} | Synechococcus sp. (strain WH7803) | Cytoplasm |
| A5GW21_SYNR3 | Ribonucleases G and E {GENE:Name=cafA; OrderedLocusNames=SynRCC307_2177} | Synechococcus sp. (strain RCC307) | Cytoplasm |
| A6F1D9_9ALTE | Ribonucleases G and E {GENE:ORFNames=MDG893_00300} | Marinobacter algicola DG893 | Cytoplasm |
| A6F4Z3_9ALTE | Ribonucleases G and E {GENE:ORFNames=MDG893_01520} | Marinobacter algicola DG893 | Cytoplasm |
| A7K4Z1_9VIBR | Ribonucleases G and E {GENE:ORFNames=VEx25_2206} | Vibrio sp. Ex25 | Cytoplasm |
| Q015E2_OSTTA | COG1530: Ribonucleases G and E (ISS) {GENE:OrderedLocusNames=Ot07g02140} | Ostreococcus tauri | Cytoplasm |
| Q0F3G3_9PROT | Ribonucleases G and E {GENE:ORFNames=SPV1_04138} | Mariprofundus ferrooxydans PV-1 | Cytoplasm |
| Q1MQA2_LAWIP | Ribonucleases G and E {GENE:Name=rne; OrderedLocusNames=LI0771} | Lawsonia intracellularis (strain PHE/MN1-00) | Cytoplasm |
| Q1MYL8_9GAMM | Ribonucleases G and E {GENE:ORFNames=RED65_07664} | Oceanobacter sp. RED65 | Cytoplasm |
| Q1N4V7_9GAMM | Ribonucleases G and E {GENE:ORFNames=RED65_01135} | Oceanobacter sp. RED65 | Cytoplasm |
| Q2SBH2_HAHCH | Ribonucleases G and E {GENE:OrderedLocusNames=HCH_05330} | Hahella chejuensis (strain KCTC 2396) | Cytoplasm |
| Q2SK60_HAHCH | Ribonucleases G and E {GENE:Name=rne; OrderedLocusNames=HCH_02135} | Hahella chejuensis (strain KCTC 2396) | Cytoplasm |
| Q5GSE0_WOLTR | Ribonucleases G and E {GENE:OrderedLocusNames=Wbm0496} | Wolbachia sp. subsp. Brugia malayi (strain TRS) | Cytoplasm |
| Q6LMA5_PHOPR | Putative ribonucleases G and E {GENE:Name=SO4094; OrderedLocusNames=PBPRA3266} | Photobacterium profundum (strain SS9) | Cytoplasm |
| Q82UM5_NITEU | Ribonucleases G and E (EC 3.1.4.-) {GENE:OrderedLocusNames=NE1457} | Nitrosomonas europaea | Cytoplasm |
| Q8D773_VIBVU | Ribonucleases G and E {GENE:OrderedLocusNames=VV2_0290} | Vibrio vulnificus | Cytoplasm |
| Q8DCG7_VIBVU | Ribonucleases G and E {GENE:OrderedLocusNames=VV1_1453} | Vibrio vulnificus | Cytoplasm |
| Q8NN49_CORGL | Ribonucleases G and E (EC 3.1.4.-) {GENE:OrderedLocusNames=Cgl2364} | Corynebacterium glutamicum (Brevibacterium flavum) | Cytoplasm |
| Q8RBB1_THETN | Ribonucleases G and E {GENE:Name=CafA; OrderedLocusNames=TTE0911} | Thermoanaerobacter tengcongensis | Cytoplasm |
