# Supplementary material for: Overcoming function annotation errors in the Gram-positive pathogen Streptococcus suis by a proteomics-driven approach
Source: BMC Genomics. 2008 Dec 5;9:588. doi: 10.1186/1471-2164-9-588 (PMC2613929; doi:10.1186/1471-2164-9-588)
Supplement: Additional file 3 — Proteins with similarity to Ssu05_1371. The Word file contains a list of proteins from Gram-positive organisms showing significant similarity to Ssu05_1371 through BLAST search. All the proteins found have the cell wall-anchoring LPXTG motif. [file 1471-2164-9-588-S3.doc]

List of proteins from Gram-positive organisms showing significant similarity to Ssu05_1371 through BLAST search. All the proteins found have the cell wall-anchoring LPXTG motif.

| **Organism and strain** | **Protein name** | **Accession** | **E-value** |
| --- | --- | --- | --- |
| *Streptococcus suis* 89/1591 | surface antigen SP1 | AY864331 | 0.0 |
| [*Streptococcus gordonii* str. Challis substr. CH1](http://www.ncbi.nlm.nih.gov/Taxonomy/Browser/wwwtax.cgi?id=467705) | [LPXTG cell wall surface protein, collagen binding domain](http://www.ncbi.nlm.nih.gov/entrez/viewer.fcgi?db=protein&val=157150237) | YP_001450927 | 1.87E-42 |
| [*Streptococcus gordonii* str. Challis substr. CH1](http://www.ncbi.nlm.nih.gov/Taxonomy/Browser/wwwtax.cgi?id=467705) | [LPXTG cell wall surface protein. collagen binding domain](http://www.ncbi.nlm.nih.gov/entrez/viewer.fcgi?db=protein&val=157150237) | YP_001449429 | 1.05E-37 |
| [*Streptococcus sanguinis* SK36](http://www.ncbi.nlm.nih.gov/Taxonomy/Browser/wwwtax.cgi?id=388919) | Collagen-binding surface protein. putative | YP_001034985 | 7.97E-23 |
| [*Enterococcus faecalis* V583](http://www.ncbi.nlm.nih.gov/Taxonomy/Browser/wwwtax.cgi?id=226185) | cell wall surface anchor family protein | [NP_814992](http://www.ncbi.nlm.nih.gov/entrez/viewer.fcgi?db=protein&val=29375838) | 7.27E-21 |
| [*Streptococcus sanguinis* SK36](http://www.ncbi.nlm.nih.gov/Taxonomy/Browser/wwwtax.cgi?id=388919) | Collagen-binding surface protein. putative | YP_001034786 | 9.49E-14 |
| [*Listeria welshimeri* serovar 6b str. SLCC5334](http://www.ncbi.nlm.nih.gov/Taxonomy/Browser/wwwtax.cgi?id=386043) | LRR/LPXTG motif internalin family protein | [YP_848510](http://www.ncbi.nlm.nih.gov/entrez/viewer.fcgi?db=protein&val=116871729) | 4.69E-10 |
| [*Lactobacillus johnsonii* NCC 533](http://www.ncbi.nlm.nih.gov/Taxonomy/Browser/wwwtax.cgi?id=257314) | [cell wall-associated serine proteinase](http://www.ncbi.nlm.nih.gov/entrez/viewer.fcgi?db=protein&val=42519889) | [NP_965819](http://www.ncbi.nlm.nih.gov/entrez/viewer.fcgi?db=protein&val=42519889) | 1.24E-09 |
| [*Clostridium novyi* NT](http://www.ncbi.nlm.nih.gov/Taxonomy/Browser/wwwtax.cgi?id=386415) | putative S-layer protein (peptidoglycan anchored) | [YP_878715](http://www.ncbi.nlm.nih.gov/entrez/viewer.fcgi?db=protein&val=118444840) | 3.27E-09 |
| [*Listeria monocytogenes* EGD-e](http://www.ncbi.nlm.nih.gov/Taxonomy/Browser/wwwtax.cgi?id=169963) | putative peptidoglycan bound protein (LPXTG motif) | [NP_465702](http://www.ncbi.nlm.nih.gov/entrez/viewer.fcgi?db=protein&val=16804217) | 9.83E-08 |
| [*Enterococcus faecalis* V583](http://www.ncbi.nlm.nih.gov/Taxonomy/Browser/wwwtax.cgi?id=226185) | [collagen adhesin protein](http://www.ncbi.nlm.nih.gov/entrez/viewer.fcgi?db=protein&val=29375675) | [NP_814829](http://www.ncbi.nlm.nih.gov/entrez/viewer.fcgi?db=protein&val=29375675) | 9.83E-08 |
| [*Bacillus weihenstephanensis* KBAB4](http://www.ncbi.nlm.nih.gov/Taxonomy/Browser/wwwtax.cgi?id=315730) | LPXTG-motif cell wall anchor domain | [YP_001643862](http://www.ncbi.nlm.nih.gov/entrez/viewer.fcgi?db=protein&val=163938978) | 3.31E-07 |
| *Streptococcus pyogenes* M1 SF370 | hypothetical protein SPy_0128 | AAK33238 | 2.80E-03 |
